# Simulation of Unidirectional Ion Ejection in Miniature Four-Channel Linear Ion Trap Array

**DOI:** 10.3390/s25216701

**Published:** 2025-11-02

**Authors:** Yunfan He, Zhuoqing Yang, Yan Zhang, Yunna Sun, Jinyuan Yao, Guifu Ding

**Affiliations:** 1 School of Integrated Circuits, Shanghai Jiao Tong University, Shanghai 200240, China; hyf1825313155@163.com (Y.H.); yzhuoqing@sjtu.edu.cn (Z.Y.); dzlzhangyan@sjtu.edu.cn (Y.Z.); cecilia_sun@sjtu.edu.cn (Y.S.); jyyao@sjtu.edu.cn (J.Y.); 2 Non-Silicon Micro/Nano Integrated Manufacturing Technology Service Platform of Shanghai, Shanghai 200240, China

**Keywords:** miniature four-channel linear ion trap array, electric field simulation, structural optimization, unidirectional ion ejection, mass resolution

## Abstract

With the surging demand for dynamic, real-time, and rapid qualitative analysis of chemical components, chip-scale mass spectrometers have attracted widespread attention. Ion traps have become the preferred mass analyzer for chip-scale mass spectrometers due to their excellent analytical performance. However, the miniaturization of ion traps inevitably leads to a reduction in ion storage capacity, which in turn affects their sensitivity and dynamic range. In this study, a Miniature Four-Channel Linear Ion Trap Array (M-FLITA) with hyperbolic electrodes and a 1 mm field radius was established and optimized. Concurrently, unidirectional ion ejection was accomplished by the application of asymmetric RF voltages on M-FLITA. The results demonstrate that, in the stretched structure, the mass resolution is improved to 732, while the unidirectional ion ejection efficiency is maintained at 96%. M-FLITA demonstrates advantages in terms of high ion storage capacity and mass resolution under high ion flux conditions, providing an ideal solution for high-performance micro mass analyzers in chip-scale mass spectrometers.

## 1. Introduction

Mass spectrometers boast a number of advantages, including reduced sample consumption, accelerated analysis speeds and enhanced resolution [1,2,3,4], which have been instrumental in facilitating the accurate detection of components in complex samples, thus becoming an indispensable analytical tool in contemporary scientific research. In the context of the ongoing advancements in domains such as emergency response and on-site handling [5], deep space exploration [6], anti-terrorism [7], and food safety [8,9,10], there has been a mounting demand for the dynamic, real-time, and rapid analytical performance of mass spectrometry. The utilization of portable mass spectrometers has emerged as a pivotal research trajectory within the domain of mass spectrometry [11]. The efficacy of it in overcoming the limitations of conventional benchtop mass spectrometers is evident in its enhanced performance in terms of working environment, power consumption, and portability. This advancement offers novel opportunities for dynamic and real-time detection. In recent years, efforts have been made to miniaturize the components of mass spectrometers to the micron scale, and particular emphasis has been placed on conducting research and development through Micro-Electro-Mechanical Systems (MEMS) technology. This development has led to the emergence of chip-scale mass spectrometers, which are gradually being transitioned from laboratory concepts to practical applications and becoming a significant branch within the field of mass spectrometry analysis [12]. Jiang et al. [13] developed a “brick” size handheld mass spectrometer measuring 28 cm × 21 cm × 16 cm in 2017. The analytical performance of the system was explored in terms of detection limit, mass resolution and mass range. Reinhardt et al. [14] developed a Planar Integrated Micro Mass Spectrometer (PIMMS) system, which integrates a microwave plasma ion source, an ionization chamber, and electron/ion extraction components. In 2020, Chang et al. [15] were the first to demonstrate the integration of a gas chromatographic column and an ion trap mass spectrometer using silicon-based microfabrication techniques. Szyszka et al. [16] were the first to fabricate a miniature quadrupole mass spectrometer (MQMS) equipped with a 70 mm quadrupole using MEMS processes and 3D printing in 2024.

The ion trap mass analyzer is the preferred choice for miniaturized mass spectrometers. The continuous reduction in its size will inevitably result in a reduction in ion storage space, consequently lowering the capacity for ion storage. This has a detrimental effect on the detection sensitivity and dynamic range of mass spectrometers, and furthermore compromises the accurate quantitative analysis performance of ion traps. Multi-channel mass spectrometers have been demonstrated to be an effective approach to enhance the throughput of mass spectrometric analysis. In 2009, Kothari et al. [17] designed, constructed, and tested a four-channel multiplex mass spectrometer equipped with rectilinear ion trap (RIT) mass analyzers. The system under consideration comprised four parallel atmospheric pressure ion sources, four RIT mass analyzers, four ion optical components, and four conversion dynode electron detectors. The instrument was accommodated within a single vacuum manifold, a design that resulted in a relatively compact volume and significant reductions in cost and complexity. However, multi-channel mass spectrometers are essentially an array of combinations of multiple individual mass spectrometers, which cannot fully meet the miniaturization requirements of mass spectrometers. Ion trap arrays represent a fundamental component in the realm of mass spectrometric analysis, operating on the basis of the collaborative functioning of numerous ion trap units. The integration of multiple miniature ion traps into an array structure represents a pivotal innovation, ensuring the overall compactness of the mass spectrometer. This innovation also leads to a substantial increase in the total ion storage capacity of the ion traps, thereby enhancing detection sensitivity and expanding the dynamic range. In 2009, Li et al. [18] developed a simple ion trap array (ITA) using printed circuit board (PCB) fabrication technology, which featured high-throughput mass analysis capabilities. In 2025, Chen et al. [19] proposed a dual-layer Linear Ion Trap mass analyzer (dLIT) based on MEMS technology and a stacked structure, with built-in RITs. This study demonstrated that dLIT is an ideal instrument for MEMS-based mass spectrometric analysis. Unidirectional ion ejection represents a further method of enhancing ion detection efficiency. In comparison with the conventional bidirectional ejection technique, unidirectional ejection has the capacity to enhance detection efficiency by almost twofold, thus offering a novel methodology for the enhancement of ion trap performance. The deviation of the electric field center inside the ion trap from the geometric center can be achieved through the design of asymmetric structures or the application of asymmetric radio frequency (RF) voltages. This deviation serves to guide ions in their movement towards a single-sided electrode. In 2018, Wu et al. [20] achieved unidirectional ejection in an Asymmetric Half-Round Rod Electrode Linear Ion Trap (A-HreLIT), with a maximum detection efficiency approaching 90%. This significant advancement provides important references for the implementation of unidirectional ejection technology. In 2023, Xu et al. [21] achieved an 86% unidirectional ejection efficiency for a half-round rod ion trap (HLIT) with a 4 mm field radius through double resonant excitation, while maintaining high mass resolution.

The present laboratory is committed to the integration of miniaturized mass spectrometers in the field of non-silicon Micro-Electro-Mechanical Systems (MEMS) processes. The development of 3D-printed vibrating vane micropumps [22] and embedded electromagnetic levitation-type micromotors with a 2.5 mm radius [23] has established the basis for the advancement of chip-scale vacuum systems and the miniaturization of mass spectrometers. The present focus of the laboratory is on the research of mass spectrometers’ core devices, including miniature ion trap mass analyzers, composite-type MEMS cold ion sources and MEMS ion detectors. The objective of this research is to further promote the innovation and application of mass spectrometer miniaturization and integration technologies. In 2021, the laboratory fabricated and characterized a miniature four-channel ion trap array (MFITA) using MEMS laser etching technology. The array under consideration consists of Rectilinear Ion Traps (RITs), and its mass resolution for butyl butyrate (mass number 230) has been demonstrated to reach 324. Furthermore, analysis of air samples confirmed the consistency of the four channels within the acceptable margin of error, thereby substantiating the viability of formulating miniaturized ion trap arrays utilizing MEMS technology [24]. Furthermore, in 2025, unidirectional ion ejection was achieved in an ultra-miniature hyperbolic linear ion trap (M-HLIT) with a field radius of only 1 mm through the “electric field compensation method,” while maintaining excellent mass resolution. This provides a novel approach for the unidirectional ion ejection scheme of linear ion traps [25].

However, under the condition of a millimeter-scale (even micrometer-scale) field radius of 1 mm, even if nearly 100% unidirectional ion ejection efficiency can be achieved, single-channel miniature ion traps still have the limitation of small storage capacity. Currently, in pursuit of simplified processes, the reported multi-channel ion trap structures have been limited to Rectilinear Ion Traps (RITs) and arc-shaped ion traps. With the support of high-precision MEMS processes and 3D printing processes, arrays of hyperbolic-structure linear ion traps will generate a near-perfect quadrupolar field, further enhancing the mass analysis performance of ion traps. In order to overcome the drawbacks of low ion storage capacity and detection efficiency of micro-sized ion traps, this study innovatively proposed a unidirectional ejection type Miniature Four-Channel Linear Ion Trap Array (M-FLITA) with a field radius of only 1 mm. The adoption of high-precision hyperbolic electrodes supported by MEMS technology, in conjunction with the precise adjustment of the electric field within the trap through the design of an asymmetric voltage, has been demonstrated to achieve unidirectional ion ejection while maintaining high-quality resolution. A systematic comparison was conducted between this M-FLITA and traditional unidirectional ejection ion traps and ion trap arrays. The subsequent analysis focused on the optimization of structural and electrical parameters after setting ion ejection slots. It has been demonstrated that this configuration can enhance the ion storage capacity and mass resolution of the ion trap without the necessity of applying supplementary excitation voltage. Concurrently, it can attain the same ion detection efficiency as a dual detector under a single detector condition, providing an innovative solution for high-performance mass spectrometry analyzers in chip-level mass spectrometers.

## 2. Materials and Methods

### 2.1. Structure of M-FLITA

**Figure 1:**
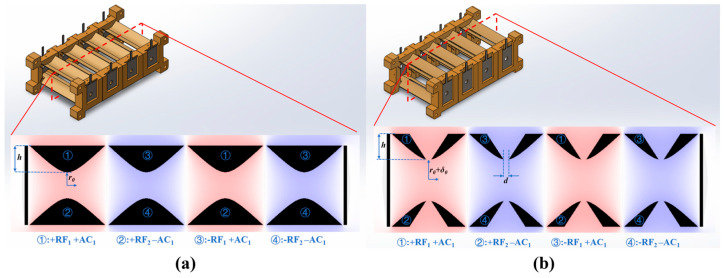
M-FLITA cross-section diagrams and voltage regulation modes: (**a**) ideal structure, (**b**) stretched structure.

The schematic diagram illustrating the overall structure of the Miniature Four-Channel Linear Ion Trap Array (M-FLITA) designed in this study is shown in Figure 1. The model was constructed exclusively to represent the electrode structure, excluding the support and end cap electrodes. This approach was necessitated by the impracticality of constructing and operating actual models. The apparatus under consideration consists of four identical hyperbolic electrode ion channels. These channels are separated by “zero-potential surfaces”, which form four mutually independent electric field regions. The cross-section diagrams and voltage regulation modes of the two structures M-FLITA are shown in Figure 1. As shown in Figure 1a, the ideal structure is characterized by the absence of ion ejection slots and bidirectional stretching compensation. Specifically, the field radius *r*_0_ is set to 1 mm, and the electrode truncation length *h* is fixed at 2 mm. The stretched structure is shown in Figure 1b. In order to compensate for the influence of ion ejection slots on the electric field components, the electrodes with ion ejection slots are translated by *δ*_0_ along the ejection direction, deviating from their original positions. The ratio *(r*_0_ *+ δ*_0_*)/r*_0_ is defined as the “stretching ratio”; the slot width of the ejection electrodes is denoted by d. Two balanced radio frequency (RF) signals with a phase difference of 180° are cross-applied to the four pairs of electrodes in order to generate a quadrupolar field. Furthermore, supplementary resonant excitation alternating current (AC) signals are applied between each pair of electrodes with a view to achieving resonant ion ejection. In both structures, RF voltages with the same phase but different amplitudes, designated *V_RF_*_1_ and *V_RF_*_2_, are applied to the upper and lower electrodes, respectively. The asymmetric voltage ratio is defined as Δ*V* = (*V_RF_*_1_ − *V_RF_*_2_)/*V_RF_*_2_. The precise regulation of the electric field is achieved by applying asymmetric voltages to the upper and lower electrodes, thereby enabling unidirectional ion ejection.

### 2.2. Electric Field Calculation and Ion Trajectory Analysis

It is a commonly held belief that the performance of an ion trap is contingent on its internal electric field distribution [26,27]. In a linear ion trap, radio frequency (RF) voltages are applied to the four electrodes. It is assumed that the electrode surfaces of the ion trap extend infinitely in space. The potential at any point (*x*, *y*) inside the trap is, therefore, expressed as follows:

(1) $$\varphi (x,y)=\frac{\varphi_{0}}{{2r_{0}}^{2}}(x^{2}-y^{2})$$

where *φ*_0_ is the potential difference generated by the voltage applied to the hyperbolic electrodes, and *r*_0_ is the field radius. The equation of motion of ions in the trap is:

(2) $$m\frac{d^{2}x}{dt^{2}}+\frac{2e}{{r_{0}}^{2}}\left(U+V\cos\Omega t\right)x=0$$

This equation can be transformed into the Mathieu equation form in order to facilitate the identification of the solution. This process results in the determination of two ion motion confinement constants, *a_μ_* and *q_μ_*, and consequently, the solution to the stability region is achieved. It is important to note that there are numerous non-ideal factors in the ion trap (e.g., electrode truncation, ion ejection slots) that have the capacity to cause quadrupolar field distortion. It has been demonstrated through rigorous research that the distribution of electric fields can be adequately characterized by the superposition and weighting of high-order multipole fields [28]. Furthermore, the internal potential distribution can be expressed as follows:

(3) $$\varphi \left(x,y\right)=V_{RF}Re\left[\sum_{n=0}^{\infty }A_{n}{\left(\frac{x+iy}{r_{0}}\right)}^{n}\right],\;n\in N$$

where *V_RF_* is the amplitude of the radio frequency (RF) voltage, *Re* denotes the real part of a complex value, r0 is the field radius, and *An* is the dimensionless amplitude of the 2n-pole field. Ions confined within a quadrupolar field oscillate in a simple harmonic manner, and ions exhibiting distinct mass-to-charge ratios (*m*/*z*) demonstrate a specific secular frequency during their motion. In the case of resonant excitation, an auxiliary alternating current (AC) signal is applied to the electrodes in the ejection direction. The adjustment of the kinetic energy and secular frequency of ions is achieved by means of scanning the amplitude of the RF voltage. When the secular frequency of ion motion matches the frequency of the auxiliary AC signal, the ions resonate—their motion amplitude increases rapidly, enabling ejection from the trap. The introduction of high-order fields has been demonstrated to induce nonlinear effects in ion motion within the ion trap. The nonlinear resonant potential energy has been demonstrated to alter the resonant excitation conditions of ions, thus degrading the performance of the linear ion trap mass analyzer. This phenomenon can be intuitively deduced from a decrease in mass resolution. Nevertheless, the presence of high-order fields does not invariably have a detrimental effect. Research has demonstrated that a suitable amalgamation of electric field components can rectify the performance deficit triggered by such nonlinear effects and even enhance resolution [29]. It is imperative to undertake a comprehensive investigation into the internal electric field, with the objective of establishing an appropriate internal high-order electric field distribution.

The calculation of ion trajectories is analogous to the integration solution process of the second-order Mathieu partial differential equation under a specified electric field condition. This process comprises two distinct steps: the calculation of the electric field and the analysis of the ion trajectory. The division of space into grid points is achieved through the implementation of numerical simulation and the finite difference method (FDM). The spatial potential distribution is obtained by solving the Laplace equation, and then the ion trajectories are calculated using the Runge–Kutta algorithm. In this study, SIMION 8.0 was utilized to establish the structural model of M-FLITA and generate potential array files. The potential array files of M-FLITA were imported into PAN33 software, which extracted samples from the potential array generated on the field radius ring, utilizing it as the boundary condition of the Laplace equation, and calculating the internal multipole field parameters. Concurrently, the prospective array files were imported into the simulation software AXSIM2016 to simulate ion trajectories and mass spectra [30]. It is imperative to acknowledge that, in order to accurately analyze the influence of ion number on ion trap performance, the space charge effect between different ions must be taken into consideration in AXSIM2016. To facilitate an intuitive performance comparison with single-channel ion traps, the mass-to-charge ratios *m*/*z* of the sample ions to be tested were set to 117, 119, and 121, with 100 ions for each *m*/*z* value [31]. The total number of ions observed in this experiment was consistent with the number of ions reported in single-channel ion trap simulations. The total number of ions is 300, which are divided into four ion trap channels, with 75 ions allocated to each channel. The initial positions of the ions were randomly distributed in proximity to the center of the storage area of the four ion channels. In this study, the classical hard-sphere collision model was employed to simulate the process. The collision gas utilized was helium, with a pressure of 0.133 Pa and a temperature of 300 K. The mass resolution of ion ejection was calculated.

(4) $$R=\;\frac{m}{∆m}$$

where *m* is the mass-to-charge ratio of the ion, and Δ*m* is the Full Width at Half Maximum (FWHM) of the mass spectral peak.

## 3. Results and Discussion

### 3.1. Performance of the Ideal Structure M-FLITA

#### 3.1.1. Mass Resolution of the Ideal Structure M-FLITA

**Figure 2:**
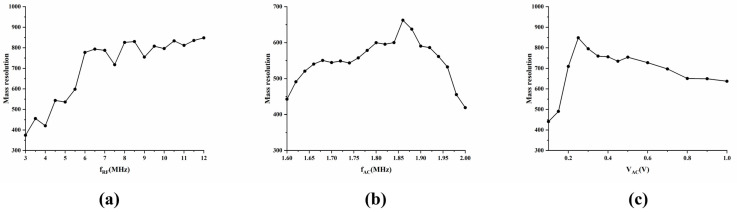
Relationship between (**a**) f_RF_, (**b**) f_AC_, (**c**) V_AC_, and bidirectional ejection mass resolution in ideal structure M-FLITA.

This study investigates the impact of electrical parameters on the analytical performance of the ideal structure M-FLITA. During the simulations, the radio-frequency (f_RF_) frequency ranged from 3 MHz to 12 MHz with an interval of 0.5 MHz, and the alternating current frequency (f_AC_) was consistently 1/3 of the f_RF_ (under the 1/3 frequency division condition). The relationship between mass resolution and f_RF_ is illustrated in Figure 2a. When the f_RF_ ranged from 3 MHz to 6 MHz, the mass resolution exhibited a rapid increase with increasing frequency, reaching 777 at 6 MHz. Thereafter, the growth rate of mass resolution tended to flatten. The optimal mass resolution was achieved when the RF was set to 10.5 MHz. However, an elevated RF will substantially augment the design complexity of the RF power supply and the system power consumption. Concurrently, the volume of the power supply will also escalate in proportion—this is remarkably disadvantageous for the miniaturization design of the mass spectrometer. Consequently, while ensuring high mass resolution, the RF should be minimized as much as possible. This approach enhances the mass resolution performance of the M-FLITA while concomitantly reducing its volume. Subsequent studies primarily adopted an RF signal frequency of 6 MHz to ensure the feasibility of the mass spectrometry system.

The alternating current (AC) frequency is a critical factor in determining the Mathieu parameter *q* of ions during the ejection process from the ion trap. A study revealed that the highest mass resolution is typically achieved when the *q* value ranges from 0.78 to 0.83 [32]. Under these conditions, the frequency of the AC voltage is approximately one-third of the radio-frequency (RF) voltage frequency. The influence of the frequency ratio of AC to RF signals (f_AC_/f_RF_) on mass resolution is demonstrated in Figure 2b, which focuses on the scenario where the RF is fixed at 6 MHz. In the simulation, the f_AC_ ranges from 1.6 MHz to 2.0 MHz with a step size of 0.02 MHz. The simulation results indicate that the optimal mass resolution is consistently achieved when the f_AC_ frequency is approximately 1.86 MHz. The findings suggest that for micro ion traps with an exceptionally diminutive radius, the resonant excitation frequency must be marginally lower than the 1/3 frequency division documented for conventional-sized ion traps to attain the optimal resonant excitation effect.

The alternating current (AC) voltage is a critical parameter for ions to achieve successful ejection under the resonant state. The variation in mass resolution of the ideal structure M-FLITA as a function of AC voltage is shown in Figure 2c. A series of simulations was conducted, with the AC voltage (V_AC_) ranging from 0.1 V to 1.0 V at a step size of 0.1 V. The results demonstrate that ions initially exhibit stable simple harmonic motion in the quadrupolar field. When the secular frequency of the ions aligns with the resonant excitation frequency, the ions can instantaneously and efficiently absorb energy, thereby achieving resonant ejection. When the AC voltage is 0.25 V, the mass resolution for bidirectional ejection reaches a peak of 848. When the AC amplitude is lower than the optimal value, ions cannot obtain sufficient energy instantly for ejection, leading to a significant decrease in mass resolution. Conversely, when the AC amplitude is excessively high, the mass resolution for bidirectional ion ejection undergoes a gradual decrease as the AC amplitude increases. Ions acquire high energy prior to reaching the ejection time point, thereby substantially augmenting their motion amplitude. Concurrently, the interference effect of intermolecular forces between ions concomitantly escalates, exerting a deleterious effect on mass resolution performance. The results indicate that under the condition of the same total number of ions, the bidirectional ejection mass resolution performance of the M-FLITA with an ideal structure improves by 47.2% compared with that of the single-channel hyperbolic linear ion trap. This finding provides substantiation for the hypothesis that the M-FLITA exhibits superior performance in terms of mass resolution.

#### 3.1.2. Unidirectional Ion Ejection of the Ideal Structure M-FLITA

**Figure 3:**
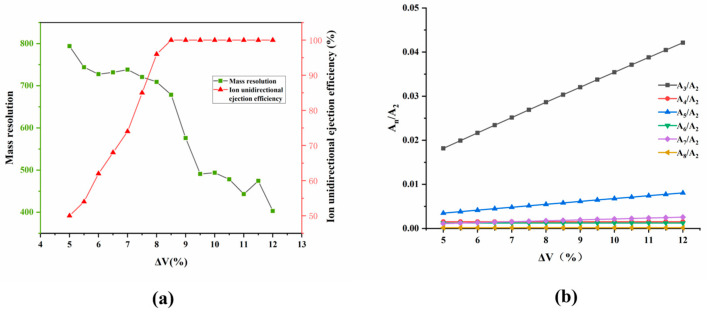
Relationship between Δ*V* and (**a**) unidirectional ejection efficiency and mass resolution, (**b**) electric field component, in ideal structure M-FLITA.

Unidirectional ion ejection simulations were performed on the optimized ideal structure M-FLITA. The radio-frequency (f_RF_) frequency was fixed at 6 MHz, the alternating current frequency (f_AC_) was fixed at 1.86 MHz, and the amplitude of the AC voltage (V_AC_) was carefully adjusted until all ions could be ejected from the ion trap. During the course of the simulations, the asymmetric voltage ratio, designated as ΔV, was varied from 5% to 12% at a step size of 0.5%. The relationships between the ion unidirectional ejection efficiency, mass resolution, and Δ*V* are shown in Figure 3a. The relationship between the electric field components and Δ*V* is presented in Figure 3b. For the ideal structure M-FLITA, when Δ*V* < 7%, the ion unidirectional ejection efficiency was relatively low (less than 70%), whereas the mass resolution could be maintained at a high level. This phenomenon can be attributed to the low content of higher-order fields and the high purity of quadrupolar fields under these conditions. When Δ*V* > 8.5%, there was a substantial increase in ion unidirectional ejection efficiency. This will inevitably lead to distortion of the electric field, resulting in an increase in the content of higher-order fields and a decrease in the quality resolution. Despite the persistent dominance of the quadrupolar field component during this process, and the relatively negligible intensity of the other field components, their effects could not be disregarded. As Δ*V* increased, the odd-order field components (A_3_, A_5_, and A_7_) exhibited a substantial increase, while the even-order field components (A_2_, A_4_, and A_6_) remained relatively constant. Consequently, the proportion of odd-order fields in the ion trap can be optimized by adjusting Δ*V*, thereby achieving an optimal balance between mass resolution and unidirectional ejection efficiency. When Δ*V* = 8.5%, both the ion unidirectional ejection efficiency and mass resolution were able to maintain favorable performance: the ion unidirectional ejection efficiency reached 100%, and the mass resolution was maintained at 678.

### 3.2. Performance of the Stretched Structure M-FLITA

#### 3.2.1. Mass Resolution of the Stretched Structure M-FLITA

In practical applications, it is necessary to open ion ejection slots on the ejection electrodes of the ion trap, and ion detectors are used to collect and count the ejected ions. The existence of ion ejection slots has been demonstrated to affect the internal electric field distribution, manifesting specifically as a reduction in the quadrupolar field content and an increase in the higher-order field content. This phenomenon has been observed to result in a degradation in the mass analysis performance of the ion trap. This issue can be addressed by adopting bidirectional “stretch,” which involves optimizing the proportion of electric field components by adjusting the distance between the electrodes in the ion ejection direction and the center of the ion trap. Consequently, this study developed and examined the stretched structure of the M-FLITA, as depicted in the ideal structure. In consideration of the practical, high-precision fabrication requirements of MEMS, the slot width d was set to 300 μm, and the stretching ratio (*r*_0_ + *δ*_0_)/*r*_0_ was varied from 1.0 to 2.0 with a step size of 0.1. All other structural and electrical parameters remained consistent with those previously described. In instances where the slot design was adopted without incorporating the “stretch” structure, a substantial decline in mass resolution was observed. When (*r*_0_ + *δ*_0_)/*r*_0_ was in the range of [1.0, 1.6], the mass resolution exhibited a rapid upward trend. This finding is consistent with the established stretch distance of single-channel ion traps, wherein both demonstrate optimal mass resolution when (*r*_0_ + *δ*_0_)/*r*_0_ = 1.6.

**Figure 4:**
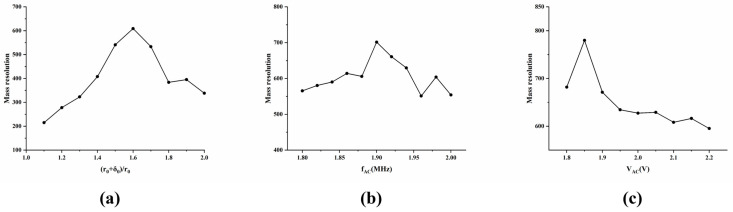
Relationship between (**a**) (*r*_0_ + *δ*_0_)/*r*_0_, (**b**) f_AC_, (**c**) V_AC_, and bidirectional ejection mass resolution in stretched structure M-FLITA.

As with the ideal structure M-FLITA, the radio frequency (f_RF_) was maintained at 6 MHz, and the AC signal was further optimized. The relationships between the bidirectional ejection mass resolution of the stretched structure M-FLITA and the AC frequency, as well as the AC voltage, are demonstrated in Figure 4b and Figure 4c, respectively. When the AC frequency (f_AC_) was 1.9 MHz and the AC voltage (V_AC_) was 1.85 V, the bidirectional ejection mass resolution reached its maximum value (779). This value was equivalent to 92% of the bidirectional ejection mass resolution of the M-FLITA with the ideal structure. Concurrently, the mass resolution performance was enhanced by 41.4% in comparison with the single-channel hyperbolic linear ion trap [31]. This finding serves to further substantiate the preeminence of the M-FLITA in terms of mass resolution under conditions involving an equivalent number of ions.

#### 3.2.2. Unidirectional Ion Ejection of the Stretched Structure M-FLITA

**Figure 5:**
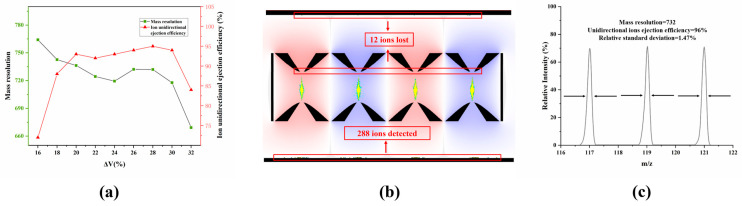
(**a**) Relationship between unidirectional ejection efficiency and mass resolution versus Δ*V*, (**b**) unidirectional ion ejection distribution diagram, (**c**) mass spectrum, in stretched structure.

As with the method for unidirectional ion ejection of the ideal structure M-FLITA, the electrical parameters were set to the optimized values described in Section 3.2.1. Then, the amplitude of the alternating current (AC) voltage was carefully adjusted until all ions could be ejected from the ion trap. During the course of the simulations, the asymmetric voltage ratio Δ*V* was varied from 16% to 32% at a step size of 2%. The relationships between the ion unidirectional ejection efficiency, mass resolution, and ΔV are illustrated in Figure 5a. In the case of the stretched structure M-FLITA, an increase in ion unidirectional ejection efficiency was observed as the Δ*V* increased, with a threshold of 28% ΔV. When Δ*V* > 28%, the high content of higher-order field components in the ion trap led to severe electric field distortion, resulting in severe ion annihilation at the ejection slots and a decrease in ion unidirectional ejection performance. Concurrently, the mass resolution exhibited a gradual decline, indicative of a continuous decrease in mass. In summary, a satisfactory balance between unidirectional ejection efficiency and mass resolution was achieved when Δ*V* = 28%: the unidirectional ejection efficiency reached 96% (shown in Figure 5b, where the green, blue and yellow dots represent the trajectories of ions with three different *m/z*), and the mass resolution reached 732. The mass spectrum is shown in Figure 5c, in which 100% corresponds to an ion intensity of 500. The relative standard deviation (RSD) among the ion resolutions of the three *m*/*z* values is only 1.47%, indicating excellent ion ejection stability. A comparison of the unidirectional ejection performance of the M-FLITA with the ideal structure reveals that the ion unidirectional ejection efficiency decreased by a mere 4%, while the mass resolution exhibited an increase of 8%. The four-channel storage capacity of the ion trap is advantageous in that it enables high unidirectional ejection efficiency and high mass resolution, even under conditions with the same number of ions. This result is significantly higher than that of the single-channel ion trap with stretch compensation under the same field radius.

#### 3.2.3. Influence of *m*/*z* and Impurity Ions

**Figure 6:**
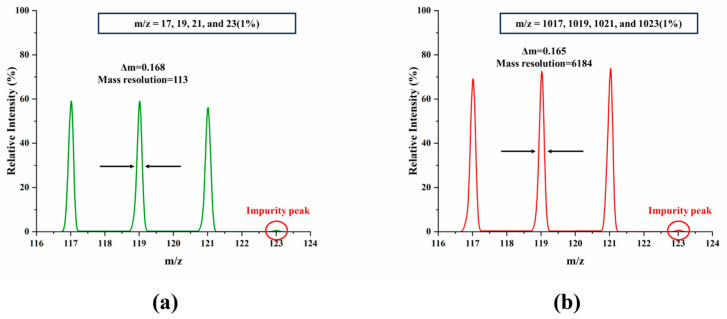
Mass spectra of different *m*/*z* ranges: (**a**) *m*/*z* = 17, 19, 21, and 23 (1%), (**b**) *m*/*z* = 1017, 1019, 1021, and 1023 (1%).

To investigate the analytical characteristics of M-FLITA further, this study simulated the mass resolution for ions with different *m*/*z* values. The first group included *m*/*z* ions of 17, 19, and 21, and the second group included *m*/*z* ions of 1017, 1019, and 1021. Figure 6a,b displays the corresponding mass spectra and demonstrates that M-FLITA maintains excellent mass resolution performance across a common *m*/*z* range. The calculation results demonstrate that high *m*/*z* (1019) ions exhibit superior performance in terms of mass resolution in comparison to low *m*/*z* (19) ions. Furthermore, 1% impurity ions (*m*/*z* = 23 and *m*/*z* = 1023) were incorporated into each of the two simulations. The findings indicated that M-FLITA exhibited the capacity to discern the presence of trace impurity ions, as evidenced by a marginal response in the mass spectrum peaks. This observation suggests that M-FLITA possesses a degree of sensitivity to trace impurity ions.

### 3.3. Ion Storage Capacity

**Figure 7:**
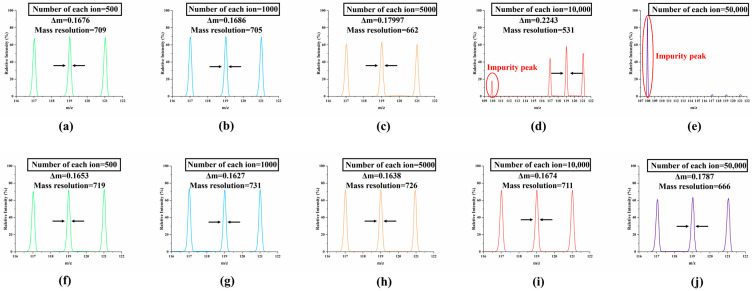
Mass spectrums at different ion fluxes (**a**–**e**): single-channel ion trap (**f**–**j**): M-FLITA.

As demonstrated by the aforementioned simulations, the unidirectional ejection M-FLITA exhibits clear advantages in terms of mass resolution performance. However, these simulations do not intuitively reflect the advantages brought by changes in its internal ion storage capacity. In this section, the number of ions stored inside the trap was adjusted to observe the performance advantages of M-FLITA in mass resolution when the number of ions increases significantly. The *m*/*z* of the three types of ions was maintained at 117, 119, and 121. The number of each type of ion was increased to 500, 1000, 5000, 10,000, and 50,000, respectively, and these ions were evenly distributed among the four ion channels. The structural and electrical parameters exhibited consistency with the previously optimized results. As illustrated in Figure 6, the mass spectra are observed to vary in accordance with the quantity of ions present. As illustrated in Figure 7a–e the mass spectra of the single-channel ion trap are depicted, with the “electric field compensation method” serving as the underlying principle. In contrast, Figure 7f–j presents the mass spectra of the M-FLITA.

The result indicates that under conditions of minimal ion flux, there is no substantial discrepancy in the mass resolution performance of the two structures. However, under conditions of high ion flux, the single-channel ion trap has been observed to exhibit uncontrolled ion ejection, a phenomenon attributed to the space charge effect. This phenomenon is primarily attributable to the elevated ion density within the trap, which leads to intense collisions between ions. These collisions result in the premature expulsion of ions from the controlled region. When the number of each type of ion reaches 5000 (shown in Figure 7c), the mass spectral peaks undergo a substantial decrease, and the mass resolution undergoes a nearly 10% reduction. When the number of each type of ion reaches 10,000, as shown in Figure 7d, unwanted peaks emerge. Concurrently, the mass spectral peaks of the target ions undergo a precipitous decline, resulting in a mass resolution that is a mere 531. When the number of each type of ion reaches 50,000, only a minute proportion of ions can be successfully ejected from the electric field according to their mass-to-charge ratios; the majority of ions cannot be confined to the center of the electric field, and the mass resolution is nearly indistinguishable (shown in Figure 7e). In contrast, the M-FHLITA has been demonstrated to maintain excellent mass resolution even under conditions of high ion flux. This finding suggests that the incorporation of an array structure within the ion trap design can lead to a substantial enhancement in the ion storage capacity, thereby significantly reducing the ion storage pressure within a single channel. When the number of each type of ion reaches 10,000, the fluctuation in mass resolution is only 2.9% (shown in Figure 7i). When the number of each type of ion reaches 50,000, the mass resolution undergoes a 9.0% decrease while maintaining adequate analytical performance (shown in Figure 7j). The findings substantiate the viability and superior performance of the multi-channel hyperbolic ion trap array with unidirectional ion ejection.

## 4. Conclusions

In order to address the pressing need for enhanced ion detection efficiency and augmented ion storage capacity in ion traps with millimeter-scale field radius, this study has devised a hyperbolic electrode Miniature Four-Channel Linear Ion Trap Array (M-FLITA) that boasts a 1 mm field radius. The attainment of unidirectional ion ejection was accomplished through the implementation of asymmetric radio frequency (RF) voltages. In order to provide a comprehensive evaluation of the benefits offered by M-FLITA, this study undertook an exhaustive examination of its performance, utilizing two pivotal metrics: unidirectional ion ejection efficiency and mass resolution. The performance of bi-directional ion ejection was optimized by adjusting RF and AC electrical parameters. For the ideal structure M-FLITA, the maximum mass resolution (848) was attained when the RF was 6 MHz and the AC frequency was 1.86 MHz. In the stretched structure, the stretching ratio (*r*_0_ + *δ*_0_)/*r*_0_ was further optimized to 1.6, the AC frequency was set to 1.9 MHz, and the AC voltage was carefully tuned. Under these conditions, the mass resolution could be maintained at 779. The performance of unidirectional ion ejection was optimized by adjusting the asymmetric voltage Δ*V*. In the ideal structure, 100% unidirectional ion ejection efficiency and a mass resolution of 678 were achieved. In the stretched structure, following the optimization of the electrical parameters, the unidirectional ion ejection efficiency was sustained at 96%, thereby demonstrating exceptional unidirectional ejection performance. Concurrently, the mass resolution attained 732, which marginally exceeds that of the ideal structure. Its mass resolution performance is excellent within the common *m*/*z* range, and it exhibits remarkable sensitivity to trace impurity ions. The findings suggest that, under conditions of equivalent ion counts, the mass resolution of M-FLITA exhibits a marked superiority over that of single-channel ion traps. Additionally, the M-FLITA demonstrates the capacity to sustain optimal mass resolution in conditions characterized by elevated ion flux. When the total number of ions reaches 150,000, the mass resolution undergoes only 9% decrease, enabling a substantial enhancement in the ion storage capacity of the ion trap. The unidirectional ejection characteristic of the apparatus under consideration has been demonstrated to achieve ion detection efficiency equivalent to that of the dual-detector mode when operating in the single-detector mode. This enhancement of mass resolution performance and storage capacity is a significant finding of the present study. This innovation provides a novel solution for high-performance mass analyzers in chip-scale mass spectrometers.

## Data Availability

The original contributions presented in this study are included in the article. Further inquiries can be directed to the corresponding author.
